# Digital Transformation and Local Government Response to the COVID-19 Pandemic: An Assessment of Its Impact on the Sustainable Development Goals

**DOI:** 10.1177/21582440231167343

**Published:** 2023-04-11

**Authors:** Pedro R. Palos-Sánchez, Pedro Baena-Luna, Mercedes García-Ordaz, Francisco J. Martínez-López

**Affiliations:** 1 University of Seville, Spain; 2 University of Huelva, Spain

**Keywords:** digital transformation, local governments, citizens, public sector, sustainable development goals (SDGs), attitude to COVID-19

## Abstract

This paper analyzes how Digital Transformation (DT) processes have influenced the Attitude of local governments (LGs) toward the COVID-19 pandemic and their effect on achieving the United Nations’ Sustainable Development Goals (SDGs). The data were collected from LGs in Spain (*n* = 124) through a questionnaire in which the IT skills of their workers, the DT processes, budgets, degree of regulatory compliance, and implementation of trust seals were measured, together with the IT security measures adopted. The contrast between the proposed model and the results showed that the direct influence of IT security influences the government’s attitude toward COVID-19 and DT implementing actions to achieve SDGs. The findings of this work are of great value both for the actors involved in the design and implementation of public policies and for those responsible for local governance in their objective to improve citizens’ experience of the services provided and in exceptional situations such as the one experienced as a result, of-COVID-19.

## Introduction

The public sector, especially local government (LG), is currently immersed in a period of constant transformation and uncertainty to which it must respond with radical changes to meet the needs of its citizens (Bokolo, 2021).

As has already happened in business, citizens (individuals, companies, and other actors) demand that the public sector undergoes a similar approach (Corydon et al., 2016). Such a transformation in public administration enables it to offer greater accessibility and flexibility in the provision of services to both individuals and companies (Jakob & Krcmar, 2018; Mergel et al., 2019). Information and Communication Technologies (ICTs) are changing how public services are delivered and how LGs relate to their users. Digital technology is ubiquitous in everyday life, transforming how people interact, communicate, and perform tasks (Bokolo, 2021; Frennert, 2021). This phenomenon can be extrapolated to the public administration and its relationship with citizens. Technological progress has transformed this relationship into a bidirectional one in which the development of an electronic administration plays a prominent role worldwide (Polat et al., 2013).

Numerous research studies examine the effect of collaboration on innovation success. This is the case of the review of the mediating role of social performance between cooperation and innovation performance conducted by Awan et al. (2018). The authors offer a distinctive perspective on innovation performance. For this purpose, a structural model was empirically investigated using partial least squares structural equation modeling (PLS-SEM) with 239 manufacturing firms. According to the findings of the observational study, social performance is a prerequisite for innovation outcomes. Our research supports the potential of addressing social issues to drive innovation performance.

The adoption of social performance techniques in industrial companies is expected to increase significantly over the next 10 years. A consequence of this will be that managers can promote sustainable innovation by collaborating with consumers and improving the social performance of their companies (Awan & Sroufe, 2019). For example, relational governance helps create a collaborative and trusting environment that facilitates the implementation of change and the adoption of new technologies. In addition, digital transformation (DT) involves changes in an organization’s culture and processes. Relational governance can help facilitate this process by involving all the stakeholders, helping to communicate changes, and ensuring that the organization’s objectives are met. Some recent studies have examined this aspect in export manufacturing industries (Awan, 2018).

DT in the public sector is not necessarily voluntary. It is often imposing, as LGs are forced to adopt digital innovation to meet the requirements of reforms launched at the national or supranational level (Lino et al., 2021). This was the case during the COVID-19 pandemic and the increasingly imperative need to align LG actions with the U.N.’s Sustainable Development Goals (SDGs). These SDGs, adopted in 2015 by the United Nations, promote balanced development in social, economic, and environmental sustainability (UNDP, 2015). The performance of the territories of the different EU countries has been irregular. This has resulted from the surprises and the short time to react by LGs (Hidayat et al., 2022).

The SDGs represent the commitment of world leaders to act on a more sustainable path toward inclusive and equity growth-able. Electronic government actions based on DT should favor the generation of a new paradigm in providing services through web-based functionalities and ICTs (ElMassah & Mohieldin, 2020). In response to changing expectations, the public sector is changing how it operates to improve the delivery of its services more efficiently and effectively, both in its design and in achieving its objectives. Other consequences include increased transparency, interoperability, sustainability, and citizen satisfaction (Chua et al., 2022; Mergel et al., 2019).

This paper addresses and analyzes the DT processes implemented by LGs in Spain from a double perspective: their influence on the achievement of the SDGs and the attitude of LGs and their employees during the COVID-19 pandemic. To achieve this objective, we have examined how LGs and their employees have adopted and integrated new electronic and digitalization processes into their daily tasks to effectively and efficiently interact with citizens. The proposed model was tested with the participation of 124 Spanish LGs. The results showed the direct influence of IT security on attitude toward COVID-19 and that of DT on implementing actions to achieve SDGs. This study found that DT processes in LGs were relevant in attaining SDGs and instrumental in resolving the difficulties raised in the relationship between LGs and citizens due to COVID-19.

The contrast of the influence exerted by manageable elements of the local governments themselves, such as the IT skills of their workers and the actions of IT security and DT in the context of potentially facilitating conditions for aligning efforts in moments of great uncertainty such as those experienced during COVID-19, constitutes one of the main contributions of this work.

## Theoretical Framework

### Digital Transformation

DT processes and the implementation of e-services in the public sector is a nuanced reality that is difficult to define (Kitsios et al., 2021). According to Ylinen (2021), we understand DT as an organization’s response to the changes in its environment based on the combined use of technical and technological resources through electronic devices, generating a wide range of possibilities to relate to the public. The essence of DT is based on the variety of options and technology-based solutions (Agostino et al., 2021). So, it is worth considering why some public administrations adopt these innovations, and others do not (Connolly et al., 2018; Mergel et al., 2019; Ruud, 2017). The reasons for the different speeds in implementing the necessary changes in many cases are based on unaligned strategies implemented between the various administrations, unimplemented structural reforms, lack of financing, etc. (Scupola & Mergel, 2022). Previous studies have described the challenges public administrations face as they are shaped and transformed by using ICTs to improve the services they provide to citizens (Tangi et al., 2020).

Within any organization, transformation, and change in the way it relates to its clients or users may be conditioned by the dimension of variables such as flexibility and the extent of bureaucracy it entails (Connolly et al., 2018), and these variables could be relevant when implementing, or not, proposed changes to improve services provided by public administrations (Hammi et al., 2018; Kuhlmann & Heuberger, 2021; Tangi et al., 2020). However, the aim of DT should be to increase agility and reduce the bureaucracy of organizational functions (Marino-Romero, Palos-Sánchez, Velicia-Martin, et al., 2022).

### Facilitating Conditions

In the case of a DT process in an LG, size can be fundamental to achieving the proposed objectives when providing electronic resources for citizens (Catalá & Penalva, 2020; Ingrams et al., 2020; Morales & Sussy, 2018). In most cases, the dimension of the LG in its budget (Chua et al., 2022; Connolly et al., 2018; Kuhlmann & Heuberger, 2021) will condition the creation of a specific department to lead, supervise, and control the DT processes implemented (Jakob & Krcmar, 2018). However, the direction and importance of this influence have not always been addressed and analyzed in previous studies (Luna-Reyes et al., 2020).

The link between the organization’s dimension and innovation is common in research on innovative processes and management (Kim et al., 2021). Small and medium-sized organizations may only sometimes be able to undertake the organizational transformation that will enable them to develop and take advantage of DT (Ylinen, 2021). However, as Luna-Reyes et al. (2020) pointed out, large populations are only sometimes the most suitable environments for implementing radical transformation processes. Excessive regulation and bureaucratic processes often prevent flexibility at the organizational level and among staff when introducing innovative working methods. LGs are public corporations that provide services according to their legal powers, budget, and assets (Lajtkepova, 2019). As Connolly et al. (2018) stated, economic resources positively influence the adoption of e-administration tools, favoring more significant innovation.

Citizens’ expectations regarding the public services provided by LGs and their financial management have given rise to many codes of conduct and ethical declarations to optimize financial and budgetary resources (Ponce et al., 2018). Codes of conduct have been incorporated into e-services information systems to provide information on ethical and professional issues to citizens and public employees on the implementation, services, support, and maintenance of information systems. They include aspects such as response time commitments, ethical behavior in data processing, and others that guarantee a good service (Chatterjee et al., 2021).

Trust seals have a similar objective but depend on an external entity. The possession of these seals increases user confidence and is linked to the use of good practices by the organization (Willems & Kamau, 2019). Such seals include quality standards such as ISO (International Organization for Standardization) or UNE (Spanish Association for Standardization), web security of applications and websites through SSL certificates that certify the authenticity of the certified website, and the security of transactions. Cloud seals identify cloud service providers that perform security analyses and comply with current regulations. Secure payments indicate security criteria in the amount of taxes or fees and legal compliance in terms of privacy and protection of citizens, and many others demonstrate the Web portal’s commitment to some aspect of security not included in the previous categories.

The relevance of the budgetary dimension, along with codes of conduct and trust seals and conditions LGs when it comes to accessing resources, acquiring knowledge, and implementing innovative actions that improve the services provided and the working conditions of their employees. Based on the above, we can establish the following research hypotheses:

*H1 (+): The facilitating conditions based on the budgetary dimension and codes of conduct and trust seals exert a direct and positive influence on implementing digital transformation actions in local governments.*
*H2 (+): The facilitating conditions based on the budgetary dimension, codes of conduct, and trust seals exert a direct and positive influence on the acquisition of resources and knowledge in the field of Information and Communication Technologies in local governments.*

### Information Technology Skills

The budgetary dimension of LG can limit access to and use of ICTs and public employees in performing their regular tasks (Luna-Reyes et al., 2020). This means that LGs lead in the indicators for implementation and development of electronic administration elements, while smaller governmental entities with fewer economic resources lag behind. The existence of redundant systems from previous working models and the inability to modernize resources and knowledge in IT skills exposes the limited potential for developing new DT initiatives (Mulder & Davits, 2020) that could improve the efficiency of services provided by municipalities (ElMassah & Mohieldin, 2020; Ingrams et al., 2020).

Within LGs, as in any organization, the security of information systems is a crucial element that is constantly evolving. This requires that the staff gain the knowledge and technological skills to manage the safety of the information systems used in developing their tasks (Brooks et al., 2018). Such knowledge and skills will improve the performance of their functions and help the organization to achieve the objectives it sets itself (Masilela & Nel, 2021). For example, Knowledge-Intensive Services (KIBS) are considered facilitators, coordinators, and generators of innovation for other client companies. For this reason, researchers discovered that digital capability is the determinant of DT in the economic sector. This positively affects digital resources and organizational performance (Marino-Romero, Palos-Sánchez, & Velicia-Martin, 2022).

The fundamental value of the IT skills acquired by LG personnel for the improvement of information systems security, together with the achievement of the objectives set by the public administration, led us to establish the following two research hypotheses:

*H3 (+): The acquisition and availability of knowledge and skills in using technology to perform the usual tasks of local government workers exert a direct and positive influence on the digital transformation actions implemented.*
*H4 (+): The acquisition and availability of knowledge and skills in the use of technology for the performance of the usual tasks of local government workers exert a direct and positive influence on the security of the information systems used.*

### Digital Transformation and Implementation of Sustainable Development Goals

The need for LGs to plan financially and strategically plan well in advance makes it difficult for municipalities and their staff to adapt to technological and environmental changes (Frennert, 2021). This shortfall in adaptation can lead to reduced access to technologies and resources that could improve the effectiveness and efficiency of the services aimed at citizens (Corydon et al., 2016), erode the working conditions of staff (ElMassah & Mohieldin, 2020; Frennert, 2021) as well as the availability of knowledge (Luna-Reyes et al., 2020; Mikalef et al., 2019).

Effectiveness and efficiency in the provision of public services by LGs through DT are related to the favorable alignment of the actions of public administrations with the balanced development in social, economic, and environmental sustainability advocated by the SDGs (Wysokińska, 2021; Ziadlou, 2021).

Thanks to the radical decrease in the cost of collecting, storing, and processing information (Wysokińska, 2021), DT offers LGs the potential to achieve SDGs by providing managers with valuable information to establish programs for the progress and improvement of citizenship (Mantovani-Ribeiro et al., 2021). Therefore, DT generates a favorable context for constructing sustainable communities by efficiently and sustainably managing data and digital information (ElMassah & Mohieldin, 2020).

How DT enables LGs to take actions to achieve sustainable development (in its three dimensions) led us to establish the following research hypothesis:

*H5 (+): Digital transformation actions by local governments enable the provision of public services effectively and efficiently aligned with Sustainable Development Goals.*

### Security and Attitudes Toward the COVID-19 Pandemic

One of the consequences of the DT actions carried out by the LGs has been the increase in the security measures adopted to protect the information systems and resources used in the provision of services (Mbunge et al., 2021). The increased use of online public services has led to a greater need for data protection and privacy, as well as confronting the broader challenges surrounding cybersecurity (Henman, 2020; Palos-Sánchez et al., 2022).

The importance attached by LGs to the security of their information systems, along with the increasing implementation of protocols to increase certainty and trust (Yang, 2020), has brought about an increased awareness and implementation of security measures by LG workers using digital tools and instruments. This has led the public sector to upgrade its workers’ technological and security infrastructure (Maher et al., 2020).

Information systems security has been vital during the COVID-19 pandemic (Wasswa et al., 2023) due to the increased use of technology for telecommuting, online education, virtual meeting (Palos-Sánchez et al., 2022), and virtual healthcare (Ali et al., 2023). This has meant increased exposure to potential cyber threats. In addition, pandemic-related information, such as patient health data, is highly confidential and must be adequately protected. Maintaining these information systems in the case of LGs during the pandemic was crucial. The use of these systems was intensive and from many points (Al Amin et al., 2023).

Based on the verification of the importance of the security measures adopted by LGs for the information systems used by their employees to deliver services to citizens, the following research hypothesis was established:

*H6 (+): Improvement in the security of the information systems of local governments exerts a direct and positive influence on the attitude of workers when carrying out their tasks during the COVID-19 pandemic.*

**Figure 1.**
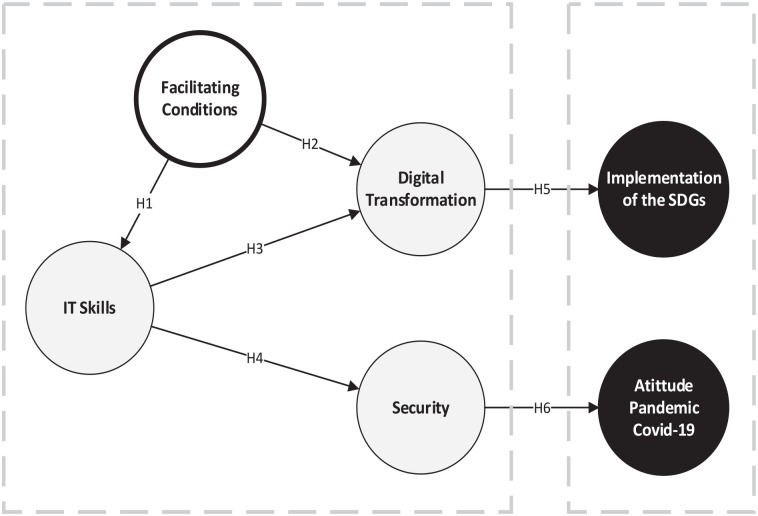
Proposed model.

The contrast of the conceptual model proposed in Figure 1 aims to identify the factors that have influenced LGs during the COVID-19 pandemic, together with those that have affected the implementation of actions to achieve the SDGs. These two dependent variables have been analyzed separately. SDG implementation was directly and positively influenced by DT, while for the other dependent variable, the attitude toward the COVID-19 pandemic, the direct and positive influence was exerted by IT security. IT security and digital transformation constructs were influenced by employees’ IT skills. The facilitating conditions affected this construct and DT, the only wholly exogenous latent variable in the proposed model.

## Materials and Methods

### Data Collection

The methodology for collecting the data and information was a questionnaire distributed to the total number of LGs in Spain in 2020 (*N* = 8,131; Table 1).

**Table 1.** Demographic Characteristics of the Municipalities.

Variable	Range	Percentage (%)
The population of the municipality	>100,000 inhabitants	59.2
	≥20,000 <100,000 inhabitants	27.5
	≥5,000 <20,000	10.8
	<5,000 inhabitants	2.5
Local government budget	<€2 million	25.8
	≥2 <€10 million	30.8
	≥10 ≤€100 million	20.8
	>€100 million	5.8
Number of employees	<50	50.0
	51–100	15.8
	101–250	10.0
	251–1,000	16.7
	>1,000	5.8

Of the total number of questionnaires sent out, 129 were returned completed. Of these, five were discarded after detecting errors in their responses.

The final study sample was *n* = 124, with a non-random convenience sampling technique.

The low response rate (1.6%) may be due to one of the following reasons: Municipalities’ usual problem lies primarily in the need for more resources and training of public employees. However, the low response rate may be owing to the need for more collaboration and coordination between different departments, municipalities, and other government agencies. Undoubtedly, this study addresses a sensitive issue because municipalities’ lack of adaptation to new technologies can affect their ability to provide adequate services, which is a fact that is not easy to recognize.

### Questionnaire and Scales

The exogenous latent variable of the proposed model and the questionnaire was:

- *Facilitating Conditions*. These were grouped into three items: the municipality government budget, measured on a scale of 4 budget intervals, as shown in Table 2. The other item used in this construct was the existence of Internet and Social Network usage rules by LGs. This variable also used an interval scale according to the degree of usage implemented by the LGs. It is essential to remember that codes of conduct in information systems are applied to offer citizens and employees ethical and professional services that cover aspects such as response time commitments, behavior in data processing, and others that guarantee a good service.

The last item used was the degree of use of information systems audits that enable trust seals. These audits depend on a second entity that audits compliance with the standards that give rise to the seal.

**Table 2.** Loadings Factors, Construct Reliability, and Validity.

Constructs	Indicators	Loadings	CA	CR	AVE
Attitude toward COVID-19 pandemic	(COV1) Information systems and digital transformation undertaken in recent years have helped them to cope better with the COVID-19 pandemic.	1.00	1.00	1.00	1.00
IT skills of employees	(ITS1) General level of IT skills	0.975	0.945	0.965	0.903
	(ITS2) IT competencies of employees	0.891			
	(ITS3) Number of IT courses	0.981			
Facilitating conditions	(FC1) Information system audits	0.970	0.958	0.973	0.922
	(FC2) Local government budget	0.949			
	(FC3) Use of rules for Internet and Social Networks	0.962			
Digital ttransformation	(DT1) Digitized information %	0.981	0.961	0.981	0.962
	(DT2) External digital processes	0.980			
Implementation of SDGs	(SDG1) SDG knowledge	0.948	0.863	0.936	0.879
	(SDG2) SDG tools implemented	0.926			
IT security	(SEC1) Importance of IT security	0.952	0.974	0.983	0.950
	(SEC2) Implementation of IT security guidelines in information systems	0.986			

*Note*. Bootstrapping 95% confidence interval using 5,000 samples. COV = COVID-19 attitude; ITS = IT skills; FC = facilitating conditions; DT = digital transformation; SDG = SDG implementation; SEC = security.

The endogenous variables proposed in the model were:

- *IT Skills*: This latent variable was measured based on three items referenced in the competencies of the Digital Competency Framework 2.0 (DigiComp; European Comission, 2021) and the General Level of IT Skills (GLITS) of LG employees. The first is with a four-level knowledge scale resulting from the previous measurement. The second item was the IT competencies of employees, and the third item referred to the number of IT-related courses taken by LG employees in the last 3 years.
- *Digital Transformation*: This latent variable was evaluated through two items: the percentage of digitized information in the LG, measured in terms of data derived from the services offered to citizens: taxes, cadastre, census, etc. The other item was the measurement on a four-level scale of the number of contracts and services digitally transformed and outsourced by LG.
- *IT Security*: This construct was measured with two items: the importance of the LG for the security of its information systems and the level of implementation of information systems security guidelines in the LG. Both were measured on a 5-point Likert scale.
- *Implementation of SDGs*: This construct was measured according to the following items: level of knowledge of the 2030 Agenda and SDGs and understanding of the LG’s computer tool used to implement SDGs.
- *Attitude toward COVID-19 Pandemic*: This variable was measured with a dichotomous item on whether the LG possessed the perception and attitude that the information systems and DT undertook in recent years had helped them to confront the COVID-19 pandemic more effectively.

### Data Analysis

Statistical analysis was carried out using PLS-SEM (PLS-PM, partial least squares path modeling). This type of analysis is well-suited for exploratory analysis. The partial least squares regression technique is considered convenient for modeling structural equations based on variance and is recommended for social sciences, specifically in the study of organizations (Sarstedt et al., 2016; Sosik et al., 2009).

## Results

### Analysis of the Measurement Model

The performance of the PLS-SEM analysis followed recommendations for reflective or B-mode constructs (Hair et al., 2019). First, the results of the loading indicators in each construct were obtained (see the first column in Table 2). The analysis of these values makes it possible to establish the level of influence of some variables on others. In all cases, these values were above 0.7. Items with loads lower than that threshold value were removed from the model.

Secondly, the construct reliability and validity were analyzed (Table 2). The Cronbach’s Alpha coefficient (CA) and the composite reliability (CR) were calculated to determine the reliability of the scales, resulting in accepted values greater than 0.7 (Sarstedt et al., 2014). These demonstrated the internal consistency of the model (Hair et al., 2012). Next, the convergent validity was evaluated by calculating the average variance extracted (AVE), yielding values higher than the recommended minimum of 0.50 (Fornell & Larcker, 1981).

Table 3 shows the results of the discriminate validity test. Discriminant validity is one of the usual criteria for evaluating scales for measuring latent constructs in the social sciences. All the indicators were sufficient since the diagonal elements were significantly larger in all cases than the multiform elements in the respective rows and columns (Fornell & Larcker, 1981; Hair et al., 2012; Ringle et al., 2015; Sarstedt et al., 2019).

**Table 3.** Discriminant Validity. Fornell-Larcker Criterion.

Constructs	COV	ITS	FC	DT	SDG	SEC
COVID-19 attitude (COV)	1.000					
IT skills (ITS)	0.927	0.950				
Facilitating conditions (FC)	0.622	0.574	0.960			
Digital transformation (DT)	0.981	0.935	0.609	0.981		
SDG implementation (SDG)	0.596	0.572	0.922	0.592	0.937	
Security (SEC)	0.624	0.580	0.959	0.611	0.904	0.975

### Analysis of the Structural Model

A structural analysis was carried out to contrast the proposed hypotheses for the relationships between the constructs of the proposed model. Firstly, the explanatory capacity of the endogenous variables was evaluated by calculating the value of *R*^2^ (Hair et al., 2019) and the model’s predictive power (Fornell & Larcker, 1981).

The first column of Table 4 shows that the model explained 39.0% of the attitude of LGs toward the COVID-19 pandemic and 35.0% of SDG implementation. The values obtained indicated a reasonable predictive value (Chin, 2010).

**Table 4.** Support for the Hypotheses.

Construct/hypothesis	*R* ^2^	Direct effect (β)	*T* statistic	*p*-Value	Support
IT skills	32.9%				
H1(+): Facilitating conditions → IT skills experience		.574	8.720	.000	Yes***
H2(+): Facilitating conditions → digital transformation		.108	2.066	.039	Yes*
H3(+): IT skills → digital transformation		.874	15.926	.000	Yes***
H4(+): IT skills → IT security		.580	8.917	.000	Yes***
Digital transformation	88.3%				
H5(+): digital transformation → SDG Implementation		.592	8.489	.000	Yes***
Attitude COVID-19	39.0%				
Security	33.6%				
H6(+): Security → attitude COVID-19		.624	9.275	.000	Yes***
SDG implementation	35.0%				

* *p* value < .05, using *t* (4,999), one-tailed test, **p value < .01, using *t* (4,999), one-tailed test, and ***p value < .001, using *t* (4,999), one-tailed test.

The second column of Table 4 shows the direct effect or standardized path coefficients, the critical values of the student’s *t*-distribution, and their corresponding *p*-value. The last column shows the interpretation based on the confidence level obtained.

Finally, the result of the model fit was evaluated according to indications by (Henseler et al., 2015). To this end, the standardized root means square residue (SRMR) was analyzed (Hu & Bentler, 1998). This expresses the average degree of these differences. The ease of fit of the model is a function of the lower SRMR. The result was 0.039, well below the SRMR recommendation <0.08 (Hu & Bentler, 1998).

## Discussion

The analysis of this research shows that it has the sufficient exploratory and predictive capacity to achieve the proposed objective since the contrast of the proposed model coincides with the findings of previous studies.

In the case of enabling conditions, the importance of the budgetary dimension in LGs is demonstrated. This result is in line with the work of Connolly et al. (2018) and Ingrams et al. (2020). They emphasize the importance of the budgetary dimension of the LGs when implementing this type of conditions, as well as their concern for the existence of codes of conduct and trust seals. This favors staff acquiring and developing ICT competencies and skills and implementing innovative actions to enable DT (Frennert, 2021; Morales & Sussy, 2018). It is true that in our work, the facilitating conditions had a minor effect on computer skills.

Another relevant finding was to test the influence of computer competencies on workers’ actions and perceptions of computer security (Luna-Reyes et al., 2020). This contrasts with a confidence level higher than 99.9%. This contrasts with a confidence level above 99.9%. Consequently, the positive impact of workers’ IT skills suggests a significant improvement in DT, in accordance with the provisions of Masilela and Nel (2021) and IT security in LGs in Spain. Continuous training and improvement of technical skills are essential for LG employees to perceive security and confidence when providing public services to citizens through electronic resources (Corydon et al., 2016).

The results on the relationship between DT and the implementation and achievement of actions related to the SDGs were consistent with those obtained with a confidence level above 99.9%. This point undoubtedly corroborates the fundamental role that all activities carried out by LGs in terms of DT have at present and in the medium term. This is not only in the technological field but also in the promotion of sustainable development from the economic, social, and environmental perspective, and is therefore aligned with the specific SDGs (Wysokińska, 2021) such as SDG 8 (Decent Work and Economic Growth), 9 (Industry, Innovation, and Infrastructure), 10 (Reduced Inequality), and 11 (Sustainable Cities and Communities).

Finally, the results on the influence of IT security on the attitude of LG’s workers toward COVID-19 were exciting, as they showed that IT security policies, in terms of importance and implementation of security guidelines (Zieba & Bongiovanni, 2022), help in the public delivery of services to citizens (Mbunge et al., 2021). Differences in employee perception may be due to the long and sometimes tortuous path for IT security to become an established element in organizations, as the following points out (Georgiadou et al., 2022). This perception that their actions and processes are protected by the IT security measures implemented by their LGs reinforced confidence in the efforts of LG employees in the execution of their work (Yang, 2020).

## Conclusions

Through the analysis of data collected from LGs in Spain, the study shows how cyber security and DT processes have affected the ability of LGs to respond effectively to the pandemic. This is valuable for the actors involved in the design and implementation of public policies and those responsible for local governance, as it will help them improve citizens’ experience of the services provided.

It is also necessary to highlight how the importance of the innovative actions developed by the LGs in the context of DT is evidenced, in terms of SDG implementation and effectiveness and efficiency, especially in the case of SDG number 8 objectives (Decent Work and Economic Growth), 9 (Industry, Innovation, and Infrastructure), 10 (Reduced Inequality), and 11 (Sustainable Cities and Communities).

Another relevant conclusion of this work is the relevance of technological security measures and processes in information systems perceived by LG employees. This will encourage a better employee attitude in situations arising from COVID-19.

### Theoretical Implications

Regarding the implications for the theory of the results of this work, it is essential to emphasize the relevance of certain (facilitating) conditions within LGs. These affect the DT and IT security processes, particularly as determinants in current challenges such as the implementation of SDGs and the situation evolving because of COVID-19.

The results of this work allow us to establish that the facilitating conditions, based on the budgetary dimension, and the protocols and trust seals (due to the direct and positive influence that they have on the actions that the LGs take) favor DT and on the potential for their workers to acquire knowledge and technical skills. These enabling conditions indirectly influence, through DT and IT skills, the achievement of the SDGs by LGs and the attitude of workers toward COVID-19.

### Practical Implications

The practical implications derived from this work for the actors and agents involved in the design and implementation of public actions and policies show the importance not only of the development of end-user policies but also that these must address previous issues that will favor the achievement of the objectives pursued.

The main benefit of this study is that it provides a detailed understanding of how digital transformation processes have influenced the attitude of LGs toward the COVID-19 pandemic and the achievement of the SDGs.

### Limitations and Future Research

Like all scientific research, aspects of this work need to be improved, such as the sample size of LG respondents concerning the total population under study. This urges caution when establishing possible extrapolations of the results to the totality of LGs. Another potential limitation is the national nature (Spain) of the LGs analyzed. Spanish LGs have some particular characteristics, such as their unitary political system in which the central government has great power over the policies and services of LGs. In contrast, LGs have greater autonomy in other European countries, such as Germany or the United Kingdom.

Another particularity is the usual more precarious financial situation than in other European countries due to the need for more financial resources and dependence on central government funds. And finally, a centralized management model, with a great weight of bureaucracy and a lack of citizen participation, while in other European countries, such as the Netherlands or Denmark, there are more decentralized and participatory management models. Future research could include municipalities in European countries.

Regarding potential future research lines, local governments’ budgetary dimensions could be analyzed comparatively in terms of the different budget sizes. Still, with similar DT and IT, security actions are undertaken.
